# Supplementary material for: Detection of helical water flows in sub-nanometer channels
Source: Nat Commun. 2024 Jun 29;15:5516. doi: 10.1038/s41467-024-49878-7 (PMC11217464; doi:10.1038/s41467-024-49878-7)
Supplement: Supplementary file 1 — Supplementary Information [file 41467_2024_49878_MOESM1_ESM.pdf]

Supplementary Information for

Detection of helical water flows in sub-nanometer channels

Pavel Zelenovskii, Márcio Soares, Carlos Bornes, Ildefonso Marin-Montesinos, Mariana Sardo, Svitlana Kopyl, Andrei Kholkin, Luís Mafra, Filipe Figueiredo

Corresponding author: zelenovskii@ua.pt

Content

1. Supplementary Notes 1
Determination of the average number of water molecules in the NT...1
Determination of water diffusion coefficients from DVS sorption data 1
Determination of volumetric rates and water fluxes 2
Estimation of D and J uncertainty...3
2. Supplementary Methods...3
3. Supplementary Figures...5
4. Supplementary Tables...10
5. Supplementary References:...11

1. Supplementary Notes

Determination of the average number of water molecules in the NT

Per one FF molecule in the NT, the number N_{WA} of water molecules adsorbed inside the nanochannels in DVS experiments can be found using the equation[1]:

$$\frac{N_{WA}}{N_{FF}} = \left(\frac{M_A}{M_0} - 1\right)\left(\frac{m_{FF}}{m_W} + \frac{N_W(0)}{N_{FF}}\right), \qquad \text{(S1)}$$

where M_A is the sample mass at the current step, and $N_W(0)$ is the initial number of water molecules in the NT. In accordance with crystallographic data obtained by single crystal X-ray scattering[2] and considering the complete filling of the first hydration layer of bound molecules, one can suppose that $N_W(0)/N_{FF} = 2$. The ratio of masses $m_{FF}/m_W = 312/18 = 17.3$ for H_2O and $m_{FF}/m_W = 312/20 = 15.6$ for D_2O.

The analysis of the absorption part of the isotherms presented in Fig. 2a in the main text showed the maximum number of the adsorbed water molecules per an FF ring of around 11.7 both for H_2O and D_2O. This value is close to that previously obtained[1] thus demonstrating the reproducibility of the experiments.

Determination of water diffusion coefficients from DVS sorption data

For a single water flow in the nanochannel, the basic equation for water vapor sorption data analysis was derived earlier[1]:

$$\frac{\partial M}{\partial t}(t) = 4C_0 \frac{D}{x_0}\left[\exp\left(-\frac{D}{x_0^2}\frac{\pi^2}{4}t\right) + \exp\left(-\frac{D}{x_0^2}\frac{9\pi^2}{4}t\right) + \exp\left(-\frac{D}{x_0^2}\frac{25\pi^2}{4}t\right)\right]. \qquad \text{(S2)}$$

Here, M is the total mass of the sample, D is the diffusion coefficient, and C_0 is water concentration outside the NTs. x_0 corresponds to the half of average length of the used NTs (Fig. S5a). Statistical analysis of FF NTs grown under ambient conditions has shown the mode length (the most probable length) of 490 μm[3], and therefore the value of x_0 = 245 μm was used in this work for the analysis. The estimation of D uncertainty due to the NTs length distribution is presented below, in the Section "*Estimation of D and J uncertainty*".

In case of m independent and immiscible water flows simultaneously occurring in the nanochannel, the resulted rate of the mass loss is a sum of mass loss rates of each flow, and Equation (S2) can be rewritten as following:

$$\frac{\partial M}{\partial t}(t)=\sum_{i=1}^{m}\frac{\partial M_i}{\partial t}=\sum_{i=1}^{m}\left\{4C_0\frac{D_i}{x_0}\right\}\left[\exp\left(-\frac{D_i}{x_0^2}\frac{\pi^2}{4}t\right)+\exp\left(-\frac{D_i}{x_0^2}\frac{9\pi^2}{4}t\right)+\exp\left(-\frac{D_i}{x_0^2}\frac{25\pi^2}{4}t\right)\right].\quad \text{(S3)}$$

Despite there are two types of bound water inside the FF NTs capable forming separate helical flows, their correlation times associated with the water mean residence time at one adsorption site and the diffusion coefficients are close and hardly distinguishable in vapor sorption experiments. Therefore, in the current study, only two flows are considered (axial and helical flows) and thus m = 2. This equation perfectly fits the experimental data at different partial pressures as it is demonstrated in Fig. 2b in the main text.

Determination of volumetric rates and water fluxes

The quantity in the curly brackets in Equation (S3)

$$V_{tot}=4C_0\frac{D_i}{x_0}\quad \text{(S4)}$$

represents a volumetric water loss rate related to the whole sample. This value, which is shortly referred to volumetric rate, is determined separately for each flow after the experimental data fitting. The volumetric rate for an individual NT, V_{NT}, is calculated as $V_{NT} = V_{tot}/N_{NT}$, where N_{NT} is the total number of NTs in the sample estimated from the sample mass M_0 after the preliminary drying:

$$M_0=m_{FF}\times N_{FF}+m_W\times N_W\,,\quad \text{(S5)}$$

where $m_{FF} = 5.18\times10^{-22}$ g is the mass of an individual FF molecule, N_{FF} is the total number of FF molecules in the sample, m_W is the mass of an individual water molecule (2.99×10^{-23} g for H_2O, and 3.32×10^{-23} g for D_2O), and N_W is the total number of water molecules in the sample. Taking in mind that initially $N_W = 2N_{FF}$ (complete filling of the first hydration layer of bound molecules), the number of FF molecules in the sample is:

$$N_{FF}=\frac{M_0}{m_{FF}+2m_W}\,.\quad \text{(S6)}$$

One average NT consists of $N_1 = 6L/c \approx 5.4\times10^6$ FF molecules, where L = 490 μm is the average length of the NTs[3], and c = 5.45 Å is the lattice cell parameter along the NT axis. This value is divided by two due to the bidirectional water release from the NT considered in the model (Fig. S5a). Therefore, the total number of NTs in the sample is:

$$N_{NT}=\frac{2N_{FF}}{N_1}=\frac{cN_{FF}}{3L}\,,\quad \text{(S7)}$$

and the volumetric water loss rate for an individual NT is:

$$V_{NT}=\frac{V_{tot}}{N_{NT}}=\frac{3LV_{tot}}{cN_{FF}}\,.\quad \text{(S8)}$$

The water flux, J, through the individual NT is the amount of water passed through the NT cross-section. It can be estimated as a ratio between the volumetric rate V_{NT} and the cross-section area S_{NT} of the nanochannel (diameter $d \approx 0.9$ nm):

$$J=\frac{V_{NT}}{S_{NT}}=\frac{4V_{NT}}{\pi d^2}\,.\quad \text{(S9)}$$

Estimation of D and J uncertainty

The calculations of the diffusion coefficient D, volumetric water loss rate V_{tot}, and water flux J include the half of the NTs length, x_0. Statistical analysis performed earlier[3] has shown that under ambient conditions the most probable length of FF NTs is 490 μm (Fig. S5b), and therefore the value of x_0 = 245 μm was used in this work for calculations. However, the NTs length dispersion, which is 240 μm (Fig. S5b), can distort the resulted values, and therefore its impact on D and J should be estimated.

The diffusion coefficient is inserted in the exponential part of Equation (S3) in the form of D/x_0^2. Therefore, x_0 dispersion influences the resulted value of D giving the uncertainty of about 50% (based on the D calculations using lower and upper values of the x_0 range). At the same time, the ratio of these values (D/x_0^2 in the exponent power or D/x_0 in the curly brackets in Equation (S3)) is less sensitive to x_0 dispersion because it is determined by the curvature of the experimental $\partial M/\partial t$ curves (Fig. 2b in the main text). Therefore, the uncertainty of V_{tot} and its derivative J is just below 1%.

2. Supplementary Methods

The molecular dynamics (MD) simulation of water diffusion through the peptide nanochannel has been done in LAMMPS package[4] and comprised in three main stages: (i) the nanochannel construction, (ii) filling the nanochannel by water molecules, and (iii) the diffusion study.

The nanochannel creation

The initial molecular structure of the NT was determined from X-ray diffraction[2] and then relaxed under periodical border conditions by DFT (see the main text). The obtained hexagonal unit cell (Fig. S6a) was replicated 50 times along the *c*-axis to get a long enough nanochannel (Fig. S6b). The resulting PDB file was converted to PSF and CRD CHARMM files using the VMD psfgen package[5] and then to LAMMPS format employing the charmm2lammps script and the CHARMM Generic Force Field (CGenFF)[6].

Filling the nanochannel

The internal cavity of the obtained FF NT was randomly filled with 1200 water molecules that correspond to 24 water molecules per ring (a helical step of the NT), the maximum capacity of the NT unit cell. The four-point TIP4P-Ew rigid water model developed for biomolecular simulations[7] implemented in LAMMPS was used.

The energy of the water filled NT was minimized using molecular mechanics prior to relaxation for 100 ps in the NVT ensemble with the Nosé-Hoover thermostat[8] maintaining the constant bath temperature at 298 K and a time coupling of 0.1 ps. Time integration was conducted using a velocity-Verlet algorithm[9] with a timestep of 1 fs. A cutoff distance of 1.2 nm was applied for both Lennard-Jones and electrostatic interactions, with the Particle-Particle Particle-Mesh (P3M) algorithm for electrostatic interactions[10].

Water diffusion study

To induce water flows in the FF NT, axial external forces of 0.63, 1.05, 1.47, and 2.09 kJ mol^{-1} $Å^{-1}$ were applied to the oxygen atoms of each water molecule, which correspond to effective pressures of 13, 22, 31, and 44 MPa, respectively. Lower pressures (c.a. 1 MPa) were unable to overcome the thermal motion of water molecules (c.a. 340 m s^{-1} at 300 K), thus leading to absence of directional flows.

The system behaviour was simulated for 7 ns under the same conditions of NVT ensemble as described above. A longer simulation for 12 ns has yield comparable results. The positions of water molecules were saved every 1 ps. After the simulation, the NT's unit cell was recentred to

facilitate further analyses (Fig. S6b). The obtained data were processed and analysed using python scripting and the MDAnalysis package[11]. The mean-squared-displacement (MSD) was computed, and the diffusion coefficient, *D*, was determined using the Stokes-Einstein equation:

$$D = \frac{1}{2d} \lim_{t\to\infty} \frac{\langle [r(t_0 + t) - r(t_0)]^2 \rangle}{t},$$

where $d = 3$ − the dimensionality of the system, and r is the position of the particle at a given time t. Initial values of D (c.a. 1000 ps) were discarded due to the ballistic behaviour of water, and the final values (c.a. 500 ps) were excluded due to the increased noise, thus following the common practice for this type of analysis[12].

Effect of the thermostat

Additional simulations were conducted to investigate the effect of the thermostat on water transport in the NT. An external force of 1.05 kJ $mol^{-1}\cdot Å^{-1}$ (effective pressure of 22 MPa) was applied and three distinct thermostat configurations were considered:

(i) NVT integrator with thermostat applied to all particles;

(ii) NVT integrator with temperature control applied based on the thermal velocity of water molecules;

(iii) NVT integrator with thermostat used to regulate the temperature of the FF NT.

For all three scenarios, thermostating the fluid reduces the applied external force inducing the flow, thus impeding the axial movement of molecules. Therefore, in line with the previous studies[13,14], thermostating the flexible NT while maintaining its net momentum to prevent drift, was found to be an optimal approach. This method ensured effective temperature control without compromising the desired flow dynamics.

Effect of water content

The effect of water content on its flow dynamics was studied by additional simulation of FF NTs with 600, 400, and 200 water molecules, which corresponds to 12, 8, and 4 water molecules per FF ring, respectively. The simulations were done under application of an external force of 0.63 kJ mol^{-1} $Å^{-1}$ (effective pressure of 13 MPa) following the aforementioned protocol.

For each system, the trajectory analysis revealed a distinct preference of water to occupy the positions near the peptide shell, where their energy is reduced due to the hydrogen bonding (Fig. S8). Moreover, the general pattern of the water distribution in the NTs with reduced filling remains the same as in the completely filled NT (Fig. S8), thus confirming the existence of helical water flows. The diffusion coefficients for such flows remains the same for each degree of filling. This is in line with the DVS experiments, demonstrating that the diffusion coefficient for helical flow weakly depends on the partial pressure (Fig. 2c in the main text).

Additionally, the amount of mobile water significantly decreased from about 25% for the fully loaded NT (24 water molecules per FF ring) to around 0.05% for the NT with 4 water molecules per ring. Although small amount of mobile water does not allow to confidently determine the diffusion coefficients for axial flows in the simulations, the deficiency of mobile water molecules is in line with the reduction of the axial water flows in partially loaded NTs observed in DVS experiments at low partial pressures (see Fig. 2d in the main text).

3. Supplementary Figures

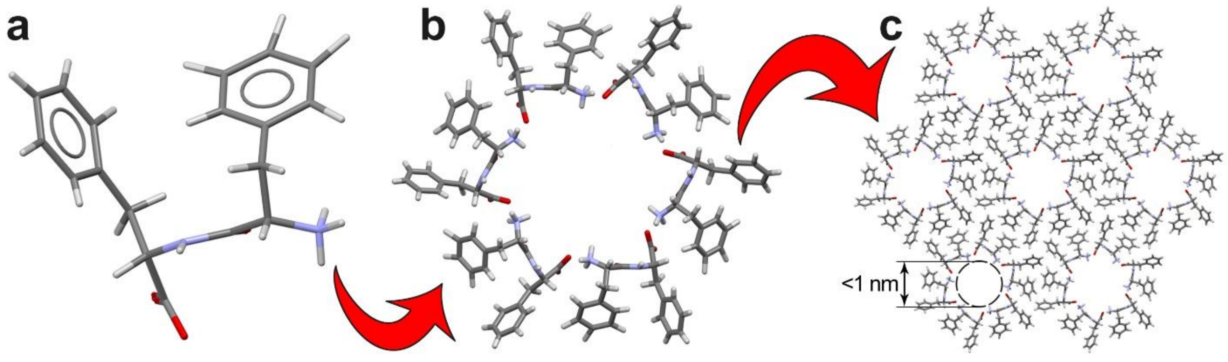

Fig. S1. The scheme of hierarchical FF NTs self-assembly. a, Individual FF monomer in the solution. **b**, Individual FF NT made of monomers held by the hydrogen bonds. **c**, The hexagonal packing of individual NTs in microbundles.

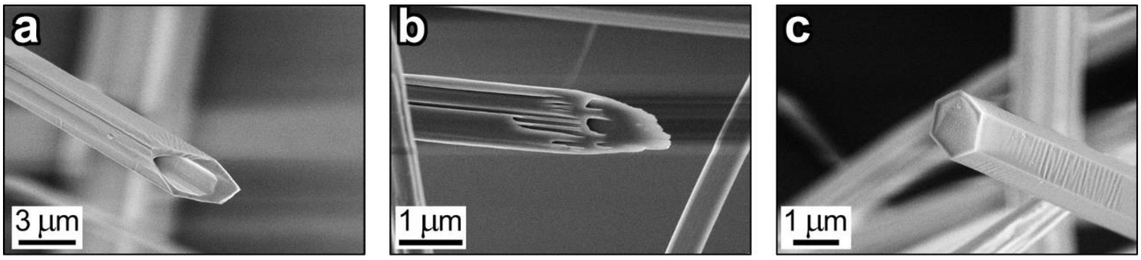

Fig. S2. SEM visualization of FF NTs bundles with and without microchannels. a, The bundle of FF NTs with one microscopic hole. **b**, The bundle with several microscopic holes. **c**, The bundle without microscopic holes.

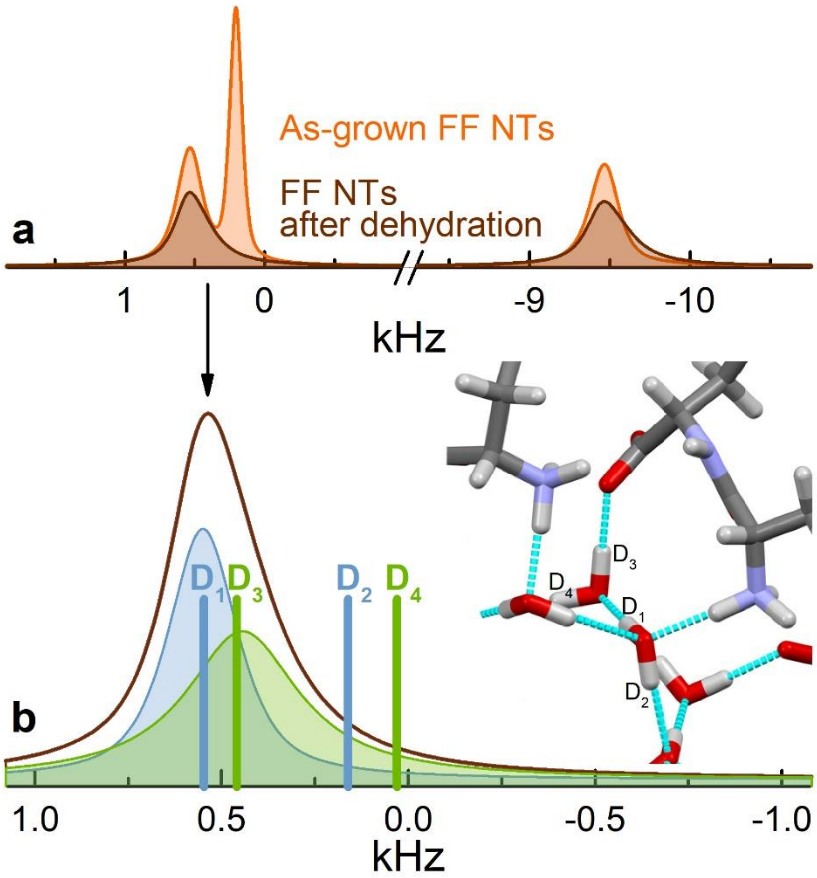

Fig. S3. Central lines in ^{2}H NMR spectra of FF NTs. a, ^{2}H NMR spectra of initial NTs and NTs after dehydration. **b**, ^{2}H NMR spectrum of the dehydrated FF NTs overlapped with deuterium chemical shifts calculated by DFT (vertical lines). The inset shows a fragment of the crystal structure with indicated location of the corresponding deuterium atoms. Cyan dotted lines denote different hydrogen bonds.

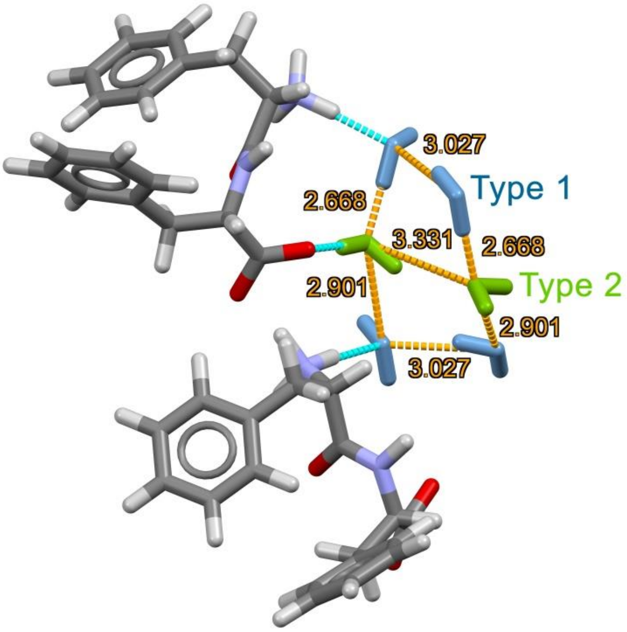

Fig. S4. The nearest adsorption sites for water molecules of Types 1 and 2 in FF NTs and distances between them. Cyan and orange lines show hydrogen bonds between different molecules.

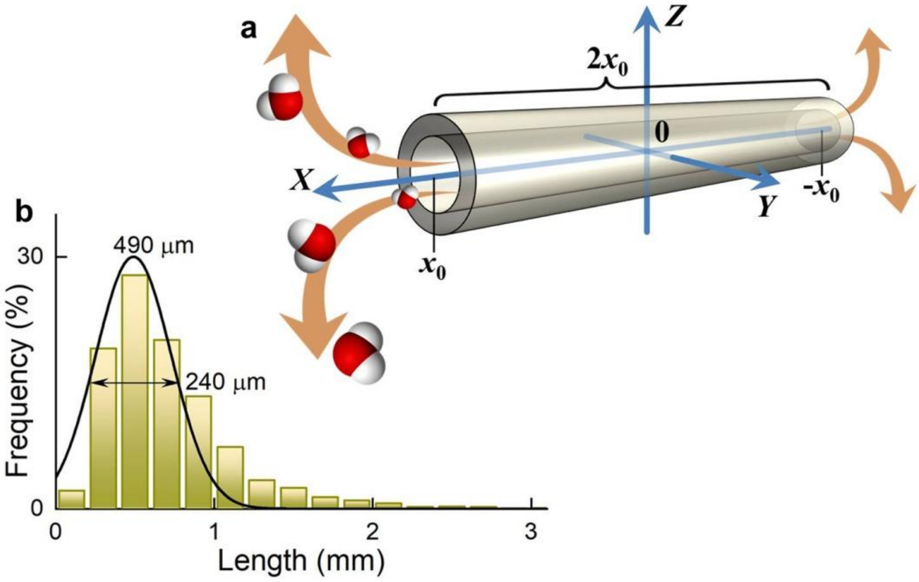

Fig. S5. Geometry of NTs used for water desorption study. a, Scheme of the FF NT used for the calculation of the diffusion coefficient. Orange arrows show the directions of the water release from the NT. **b**, Length distribution of D-FF NTs fitted to a Gaussian function[3].

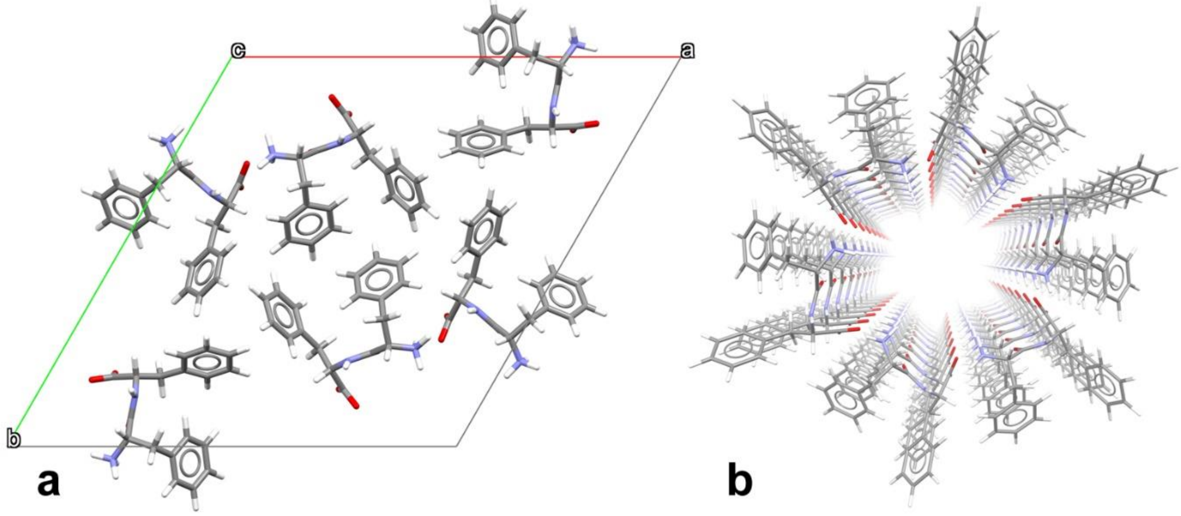

Fig. S6. The reconstructed structure of FF NT used in MD simulation. a, Initial hexagonal unit cell (space group $P6_1$) after the relaxation under periodical boundary conditions. **b,** The FF NT used for water diffusion analysis and visualization. Carbon atoms are denoted in grey, hydrogen in white, nitrogen in blue, and oxygen in red.

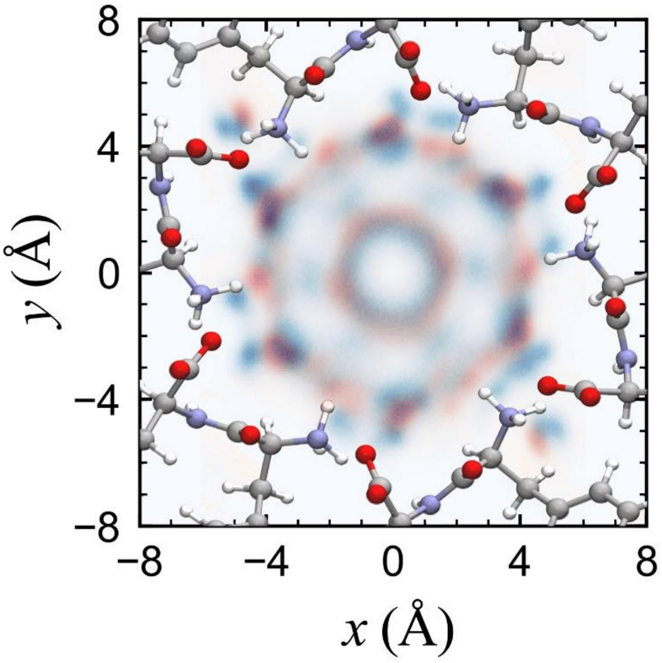

Fig. S7. Molecular dynamics simulation of water molecules distribution in the FF NT. The superposition of the coordinate projections of the oxygen (red) and hydrogen (blue) atoms of water molecules onto the nanochannel's cross-section (*xy* plane). MD simulations for 24 water molecules per FF ring and external pressure 13 MPa.

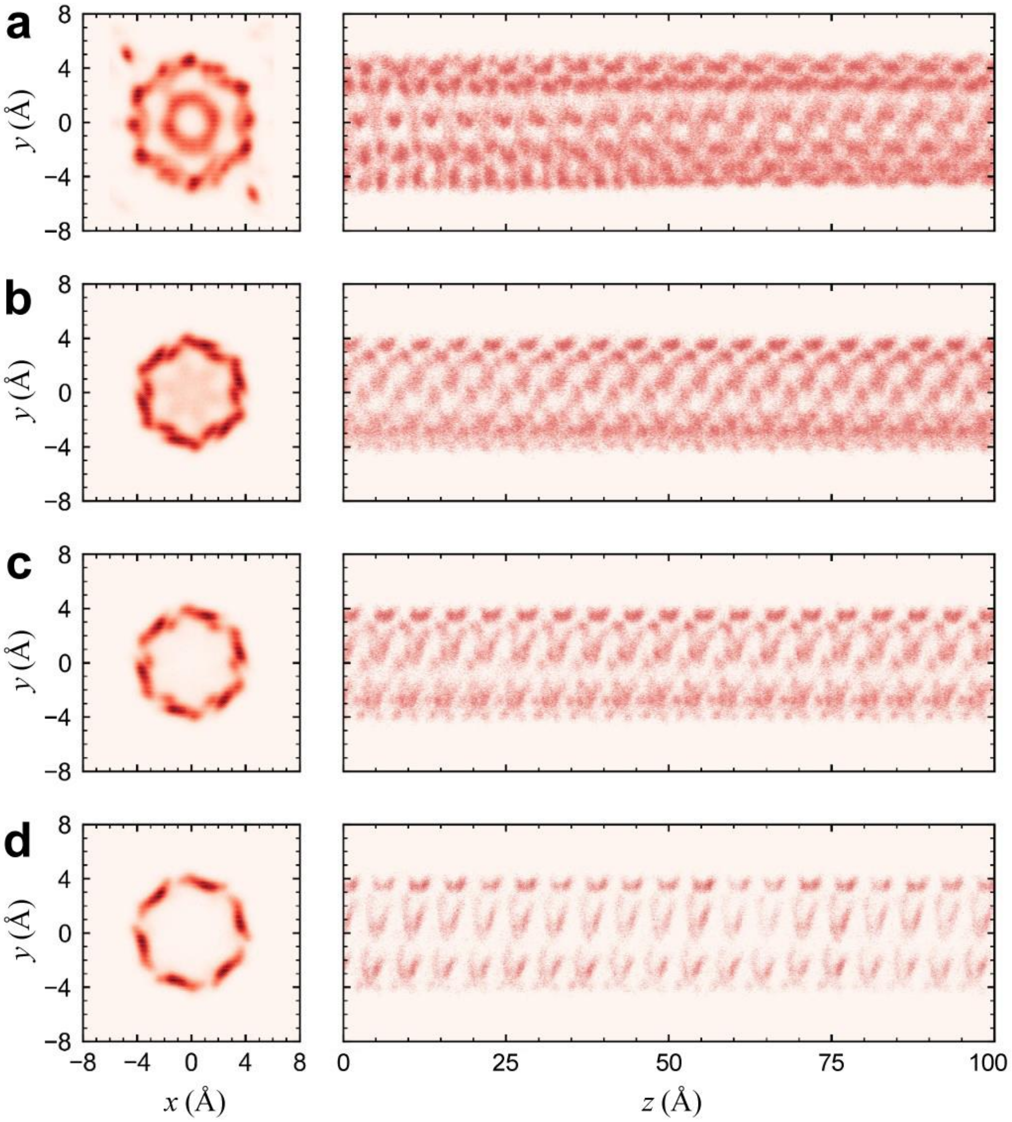

Fig. S8. Projection of the water oxygen atoms coordinates on the NT cross-sections during the flow simulation, computed for variable water content: **a,** 24, **b,** 12, **c,** 8, and **d,** 4 water molecules per FF ring. Applied external force is 0.63 kJ mol^{-1} $Å^{-1}$ (effective pressure 13 MPa).

4. Supplementary Tables

Table S1. NMR parameters of ^{2}H atoms in bound water retrieved from DFT calculations.

Water type	Attribution	Atom label	Chemical shift, ppm	Resonance frequency, kHz	C_Q, kHz
1	$D_2O\cdots NH_3^+$	D_1	8.93	0.55	181.03
		D_2	2.63	0.16	247.03
2	$D_2O\cdots COO^-$	D_3	7.48	0.46	195.55
		D_4	0.50	0.03	270.68

Table S2. Comparison of the water diffusion coefficients in nanostructured materials.

Material	D, × 10^{-10} m^2 s^{-1}	Channel size, nm	Method	Refs.
**Peptide nanotubes**				
FF nanotubes	1.3 – 3.0	<1.0	DVS measurements	1
cyclo(-WL-)$_n$	0.55 – 1.3	0.6 – 1.0	Molecular dynamics simulations	15
cyclo(-Gln-D-Ala-Glu-D-Ala-)$_4$	4.4	1.0	Molecular dynamics simulations	16
**Biological materials**				
Gramicidin A	0.28	0.5	Molecular dynamics simulations	17
Aquaporin 1	4 – 8	1.0 – 1.2	Osmotic pressure measurements	18
**Other materials**				
Nafion®	0.1 – 19.2	3.2	Pulsed gradient spin echo NMR; Computer modelling	19, 20
LTA and FAU zeolites	0.5 – 1.3	0.3 – 1.0	Sorption measurements	21
Hydroxyapatite	10	2.0 – 24.0	Molecular dynamics simulations	22
Bulk water self-diffusion	21 – 26	–	Capillary method	18, 23
Carbon nanotubes	26 – 96	1.2 – 3.5	2D diffusion–relaxation ^{1}H NMR measurements	24

5. Supplementary References:

1. Zelenovskiy, P. S., Domingues, E. M., Slabov, V., Kopyl, S., Ugolkov, V. L., Figueiredo, F. M. L. & Kholkin, A. L. Efficient water self-diffusion in diphenylalanine peptide nanotubes. *ACS Appl. Mater. Interfaces* 12, 27485–27492 (2020). doi: 10.1021/acsami.0c03658
2. Görbitz, C. H. Nanotube formation by hydrophobic dipeptides. Chem. Eur. J. 7, 5153–5159 (2001). doi: 10.1002/1521-3765(20011203)7:23<5153::aid-chem5153>3.0.co;2-n
3. Zelenovskiy, P. S., Nuraeva, A. S., Kopyl, S., Arkhipov, S. G., Vasilev, S. G., Bystrov, V. S., Gruzdev, D. A., Waliczek, M., Svitlyk, V., Shur, V. Ya., Mafra, L. & Kholkin, A. L. Chirality-dependent growth of self-assembled diphenylalanine microtubes. Cryst. Growth Des. 19, 6414−6421 (2019). doi: 10.1021/acs.cgd.9b00884
4. Thompson, A. P., Aktulga, H. M., Berger, R., Bolintineanu, D. S., Brown, W. M.¸ Crozier, P. S., in Veld, P. J., Kohlmeyer, A., Moore, S. G., Nguyen, T. D., Shan, R., Stevens, M.J., Tranchida, J., Trott, C., Plimpton, S. J. LAMMPS - a flexible simulation tool for particle-based materials modeling at the atomic, meso, and continuum scales. Comput. Phys. Commun. 271, 108171 (2022). doi: 10.1016/j.cpc.2021.108171
5. Humphrey, W., Dalke, A., Schulten, K. VMD: Visual molecular dynamics. J. Mol. Graph. 14, 33-38 (1996). doi: 10.1016/0263-7855(96)00018-5
6. Vanommeslaeghe, K., Hatcher, E., Acharya, C., Kundu, S., Zhong, S., Shim, J., Darian, E., Guvench, O., Lopes, P., Vorobyov, I., Mackerell Jr., A. D. CHARMM general force field: A force field for drug-like molecules compatible with the CHARMM all-atom additive biological force fields. J. Comput. Chem. 31, 671-690 (2010). doi: 10.1002/jcc.21367
7. Horn, H. W., Swope, W. C., Pitera, J. W., Madura, J. D., Dick, T. J., Hura, G. L., Head-Gordon, T. Development of an improved four-site water model for biomolecular simulations: TIP4P-Ew. J. Chem. Phys. 120, 9665–9678 (2004). doi: 10.1063/1.1683075
8. Martyna, G. J., Klein, M. L., Tuckerman M. Nosé–Hoover chains: The canonical ensemble via continuous dynamics. J. Chem. Phys. 97, 2635–2643 (1992). doi: 10.1063/1.463940
9. Verlet, L. Computer "Experiments" on Classical Fluids. I. Thermodynamical Properties of Lennard-Jones Molecules. Phys. Rev. 159, 98 (1967). doi: 10.1103/PhysRev.159.98
10. Hockney, R. W. and Eastwood, J. W. Particle-particle-particle-mesh (P3M) algorithms in Computer simulation using particles (CRC Press, 1988), pp 267–304.
11. Michaud-Agrawal, N., Denning, E. J., Woolf, T. B., Beckstein, O. MDAnalysis: A toolkit for the analysis of molecular dynamics simulations. J. Comput. Chem. 32, 2319-2327 (2011). doi: 10.1002/jcc.21787
12. Maginn, E. J., Messerly, R. A., Carlson, D. J., Roe, D. R., Elliot, J. R. Best Practices for Computing Transport Properties 1. Self-Diffusivity and Viscosity from Equilibrium Molecular Dynamics. Living J. Comp. Mol. Sci. 1, 6324 (2018). doi: 10.33011/livecoms.1.1.6324
13. Sam, A., Kannam, S. K., Hartkamp, R., Sathian, S. P. Water flow in carbon nanotubes: The effect of tube flexibility and thermostat. J. Chem. Phys. 146, 234701 (2017). doi: 10.1063/1.4985252
14. Bernardi, S., Todd, B. D., Searles, D. J. Thermostating highly confined fluids. J. Chem. Phys. 132, 244706 (2010). doi: 10.1063/1.3450302
15. Liu, J., Fan, J., Tang, M., Cen, M., Yan, J., Liu, Z. & Zhou, W. Water diffusion behaviors and transportation properties in transmembrane cyclic hexa-, octa- and decapeptide nanotubes. J. Phys. Chem. B 114, 12183−12192 (2010). doi: 10.1021/jp1039207

16. Engels, M., Bashford, D., Ghadiri, M. R. Structure and dynamics of self-assembling peptide nanotubes and the channel-mediated water organization and self-diffusion. A molecular dynamics study. J. Am. Chem. Soc. 117, 9151−9158 (1995). doi: 10.1021/ja00141a005

17. Chiu, S. W., Jakobsson, E., Subramaniam, S. & McCammon, J. A. Time-correlation analysis of simulated water motion in flexible and rigid gramicidin channels. Biophys. J. 60, 273−285 (1991). doi: 10.1016/S0006-3495(91)82049-5

18. Heymann, J. B. & Engel, A. Aquaporins: Phylogeny, structure, and physiology of water channels. News Physiol. Sci. 14, 187−193 (1999). doi: 10.1152/physiologyonline.1999.14.5.187

19. Zhao, Q., Majsztrik, P. & Benziger, J. Diffusion and interfacial transport of water in Nafion. J. Phys. Chem. B 115, 2717−2727 (2011). doi: 10.1021/jp1112125

20. Paddison, S. J. & Paul, R. The nature of proton transport in fully hydrated Nafion. Phys. Chem. Chem. Phys. 4, 1158−1163 (2002). doi: 10.1039/B109792J

21. Yamamoto, T., Kim, Y. H., Kim, B. C., Endo, A., Thongprachan, N. & Ohmori, T. Adsorption characteristics of zeolites for dehydration of ethanol: Evaluation of diffusivity of water in porous structure. Chem. Eng. J. 181-182, 443−448 (2012). doi: 10.1016/j.cej.2011.11.110

22. Honório, T., Lemaire, T., Tommaso, D. D. & Naili, S. Anomalous water and ion dynamics in hydroxyapatite mesopores. Comp. Mater. Sci. 156, 26−34 (2019). doi: 10.1016/j.commatsci.2018.08.060

23. Wang, J. H. Self-diffusion coefficients of water. J. Phys. Chem. 69, 4412 (1965). doi: 10.1021/j100782a510

24. Hassan, J., Diamantopoulos, G., Gkoura, L., Karagianni, M., Alhassan, S., Kumar, S. V., Katsiotis, M. S., Karagiannis, T., Fardis, M., Panopoulos, N., Kim, H. J., Beazi-Katsioti, M. & Papavassiliou, G. Ultrafast stratified diffusion of water inside carbon nanotubes; Direct experimental evidence with 2D D−T_2 NMR spectroscopy. J. Phys. Chem. C 122, 10600−10606 (2018). doi: 10.1021/acs.jpcc.8b01377
